# The embryo mosaicism profile of next-generation sequencing PGT-A in different clinical conditions and their associations

**DOI:** 10.3389/frph.2023.1132662

**Published:** 2023-03-27

**Authors:** Hadassa Campos Heiser, Natalia Fagundes Cagnin, Mariane Uehara de Souza, Taccyanna Mikulski Ali, Paula Regina Queiroz Estrada, Camila Cristina Wuaquim Dantas de Souza, Bruno Coprerski, Carmen Rubio, Marcia Riboldi

**Affiliations:** ^1^ Laboratory of Genetic Medicine, Igenomix Brasil, São Paulo, Brazil; ^2^ R&D, Igenomix SLU, Valencia, Spain

**Keywords:** PGT-A, NGS, aneuploidy, mosaicism, IVF

## Abstract

**Introduction:**

Uniform chromosome abnormalities are commonly seen in early pregnancy loss, with analyses of the product of conception suggesting the presence of mosaic autosomal trisomy in ∼10% of cases. Although chromosomal mosaicism occurs in a minority of embryos, their relative commonality and uncertainty regarding associated transfer outcomes have created discussion at both the clinical and research levels, highlighting the need to understand the clinical conditions associated with the incidence of embryo mosaicism.

**Methods:**

We took advantage of a preimplantation genetic testing for aneuploidy (PGT-A) database created from 2019 to 2022 in more than 160 in vitro fertilization (IVF) clinics in Brazil, the second-largest world market for IVF. We carried out descriptive statistical and associative analyses to assess the proportions of mosaicism associated with clinical conditions and reported incidence by chromosome, clinic origin, and biopsy operator.

**Results:**

Chromosomal analysis revealed that most mosaic aneuploidies occurred in the last three chromosomes, with 78.06% of cases having only one chromosome affected. Low mosaicism in trisomy represented the most ordinary form, followed by low mosaicism in monosomy. We identified associations between low (negatively-associated) and high mosaicism (positively-associated) and maternal age, indication (male factor and uterus/ovarian factor negatively associated with low and high mosaic, respectively), day of blastocyst development (day five has an overall better outcome), morphology grade (lower quality increased the chances of low and high mosaicism), origin (vitrified oocyte and embryo increased the rates of low and high mosaicism, respectively), and embryo sex (male embryos negatively associated with low mosaic).

**Discussion:**

With these results, we hope to foster an improved understanding of the chromosomal mosaicism linked with distinct clinical conditions and their associations in Brazil.

## Introduction

Uniform chromosome abnormalities are commonly observed in early pregnancy loss, with analyses suggesting the presence of mosaic autosomal trisomy in only 10% of the products of conception (1). Unlike uniform aneuploidy resulting from meiotic errors affecting the whole embryo, mosaic aneuploidy occurs due to a mitotic error originating in two or more cell populations with different chromosomal counts in the same embryo.

Advanced techniques for genetic screening, such as next-generation sequencing (NGS) protocols for preimplantation genetic testing for aneuploidy (PGT-A), have allowed the high-resolution visualization of the genetic material of embryonic cells and an improved distinction between uniform and mosaic aneuploidies. Notably, reported embryo mosaic rates vary from center to center (2) caused partially by differences in analysis and reporting protocols and the laboratory conditions employed for embryo biopsy (a collection of 3–10 cells from the trophectoderm). Although mosaic chromosomes occur in a minority of embryos, their relative commonality and uncertainties regarding their transfer outcome have prompted discussions at the clinical and research level, which has highlighted a need to understand any links between clinical conditions and embryo mosaicism and hence improve clinical management.

A previous study by Rodrigo. et al. that evaluated IVF cycle characteristics contributing to the incidence of mosaicism found a significant association between female age and embryo origin with mosaicism, low mosaicism probability decreased with female age but increased in the case of embryo vitrification (3). Similarly, Villanueva-Zúñiga et al. evaluated mosaicisms in young, healthy women (below 30 years old), finding that most mosaic embryos had a low chromosomal impact (4). In addition, the authors of this second study discovered a correlation between embryo quality and mosaicism where good-quality embryos displayed lower mosaicism rates than fair- and poor-quality blastocysts (4).

Our current study aimed to evaluate the influence of clinical factors on mosaicism in a sizeable Brazilian sample population (1,05,752 trophectoderm biopsies) by assessing previously reported but also exploring newly associated clinical factors. Thus, we aim to better understand the mosaic profile associated with different clinical conditions and their associations in the Brazilian population.

## Materials and methods

### Study design

The current study was a retrospective observation of trophectoderm embryo biopsies from PGT-A tests performed between Jan 2019 and September 2022 in the Igenomix Brasil laboratory (Laboratory of Genetic Medicine, São Paulo, Brazil). The trophectoderm biopsies were classified as euploid (no chromosomal abnormality observed) or aneuploid (one or more chromosomal abnormalities observed). The aneuploidies observed in the trophectoderm biopsies were individually categorized as (a) whole uniform aneuploidy (at least one aneuploidy for a whole chromosome observed), (b) segmental aneuploidy (only partial deletion/duplications above 10 Mb observed), (c) low mosaicism (one or two low mosaic degree aneuploidies without additional uniform or segmental aneuploidies observed), (d) high mosaicism (one or two high mosaic degree aneuploidies or one low and one high mosaic degree aneuploidies without additional uniform or segmental aneuploidies observed), or (e) multiple aneuploidies (a combination of two or more of the categories A, B, and C/D).

The aneuploidy categories A, B, C, and D were considered in relation to the following clinical characteristics for analysis: maternal and paternal age, embryo origin (vitrified before the trophectoderm biopsy vs. fresh, which means that it was not vitrified prior to the trophectoderm biopsy), day of blastocyst growth, embryo sex, morphology grade, chromosomes involved, indication, clinic/center of origin and biopsy operator.

### Study population and variables

The study included 1,06,777 trophectoderm biopsies from 166 clinics/centers in Brazil. Patients of maternal age ≥43 years old at the moment of the embryo biopsy and trophectoderm biopsies from ovum donation were excluded from the analysis.

The different origins of the embryos were: fresh oocyte (FO) (oocytes that have not been frozen before the trophectoderm biopsy), vitrified blastocyst (VB), vitrified oocyte (VO), and vitrified embryo (VE), before performing the trophectoderm biopsy. Clinical indications for PGT-A were (1) advanced maternal age (>37 years old) (AMA), (2) aneuploidy screening (AS), (3) male factor (MF), (4) repetitive implantation failure (IF), (5) uterus/ovarian factor (UOF), (6) recurrent pregnancy loss (PL), (7) endometriosis (EN), (8) a combination of two or more indications (MIX), (9) unknown and (10) others. Patients with abnormal karyotypes and monogenic diseases as an indication were excluded. Trophectoderm biopsies from day-5, day-6 and day-7 blastocysts were analyzed. Blastocysts were morphologically assessed using the Gardner method (Expansion: 2–6; Inner mass cell: A, B, C, and D; Trophectoderm: A, B, C, and D) (5).

### Next-generation sequencing of trophectoderm biopsies

NGS analysis from trophectoderm biopsies at day-5, day-6 and day-7 blastocysts was conducted using an Ion ReproSeq PGS Kit (Thermo Fisher Scientific, Waltham, MA, United States). The material was purified and afterwards quantified with the Qubit™ dsDNA HS Assay Kit ThermoFisher (Invitrogen, Carlsbad, CA). The processing was performed on the Ion Chef™ and Ion S5 System instruments (Thermo Fisher Scientific, Waltham, MA, United States). Ion Reporter Software (Thermo Fisher) was used for data analysis using human genome reference (hg19) (6). The samples included in this study met the minimum sequencing data quality criteria: reads per sample >70.000; MADP score (read coverage noise detected across all amplicons) <0.30; duplicated reads <30%. Samples outside these minimum quality parameters were not included in this study.

### Diagnosis of mosaicism

An embryo is considered as aneuploid when the trophectoderm biopsy presents one copy (monosomy) or three copies (trisomy) instead of the expected two copies. The level of mosaicism varies with each biopsy and may not represent the level of mosaicism of the embryo as a whole. The presence of mosaicism in a trophectoderm biopsy indicates an increased risk that the embryo is truly mosaic. Based on our in-house validation of the PGT-A test by NGS, samples are reported with a “low degree of mosaicism” (low mosaicism) in the presence of more than 30% and less than 50% of aneuploid cells in the biopsy. Samples are reported with a “high degree of mosaicism” (high mosaicism) in the presence of more than 50% and less than 70% of aneuploid cells in the biopsy (7). Biopsies with less than 30% of aneuploid cells were considered as euploid and those with more than 70% of aneuploid cells were considered as whole uniform aneuploid. For segmental aneuploidy, the size of the partial deletion or duplication must be above 10 Mb and have between 50% and 70% of segmental aneuploidy to be considered as mosaic segmental.

### Statistical analysis

All analyses were performed using R (version 4.1.2) (8). Mean, standard deviation, and proportions were used to summarize and compare the clinical characteristics of samples among the distinct types of results (low mosaic, high mosaic, euploid, whole aneuploidy, and segmental). Categorical variables (origin, day of blastocyst growth, embryo sex, morphology, and indication) used pairwise chi-squared comparisons between pairs of proportions. Continuous variables (maternal and paternal age) were compared using pairwise *t*-tests between group levels in both cases with Bonferroni corrections for multiple testing.

Logistic regression models for each of the outcomes evaluated were adjusted to prove associations between low and high mosaicism and clinical characteristics. As the variables are not independent, the models were adjusted considering all the above-mentioned variables to correct for possible confounders and access the effect of each variable independently.

Seven different models were adjusted—three with low mosaicism and three with high mosaicism as the dependent variable (y) compared to euploid and other aneuploidy categories. The seventh model was adjusted to compare low mosaicism with high mosaicism, regarding the clinical characteristics of the embryos. Thus, the seven models were: (1) low mosaic trophectoderm biopsies vs. euploid trophectoderm biopsies (*N* = 2,98,900), (2) low mosaic trophectoderm biopsies vs. segmental aneuploidy trophectoderm biopsies (*N* = 6,219), (3) low mosaic trophectoderm biopsies vs. whole aneuploidy trophectoderm biopsies (*N* = 29,133), (4) high mosaic trophectoderm biopsies vs. euploid trophectoderm biopsies (*N* = 28,180), (5) high mosaic trophectoderm biopsies vs. segmental aneuploidy trophectoderm biopsies (*N* = 4,509), (6) high mosaicism vs. whole aneuploidy (*N* = 27,423) and (7) low mosaic trophectoderm biopsies vs. high mosaic trophectoderm biopsies, where low mosaic was the outcome. All models were adjusted for maternal and paternal age, embryo origin, the day of blastocyst growth, embryo sex, morphology, and clinical indication. The odds ratio for each coefficient and the confidence interval at 95% were calculated to analyze the effect of each characteristic on low and high mosaicism.

Finally, to confirm the association of the variables against possible collinear effect, we run the same regression models but this time adding interactions to it. We tested interactions of maternal age with indication and biopsy day with morphology (expansion, inner cell mass and trophectoderm).

## Results

### Study population

**Figure 1:**
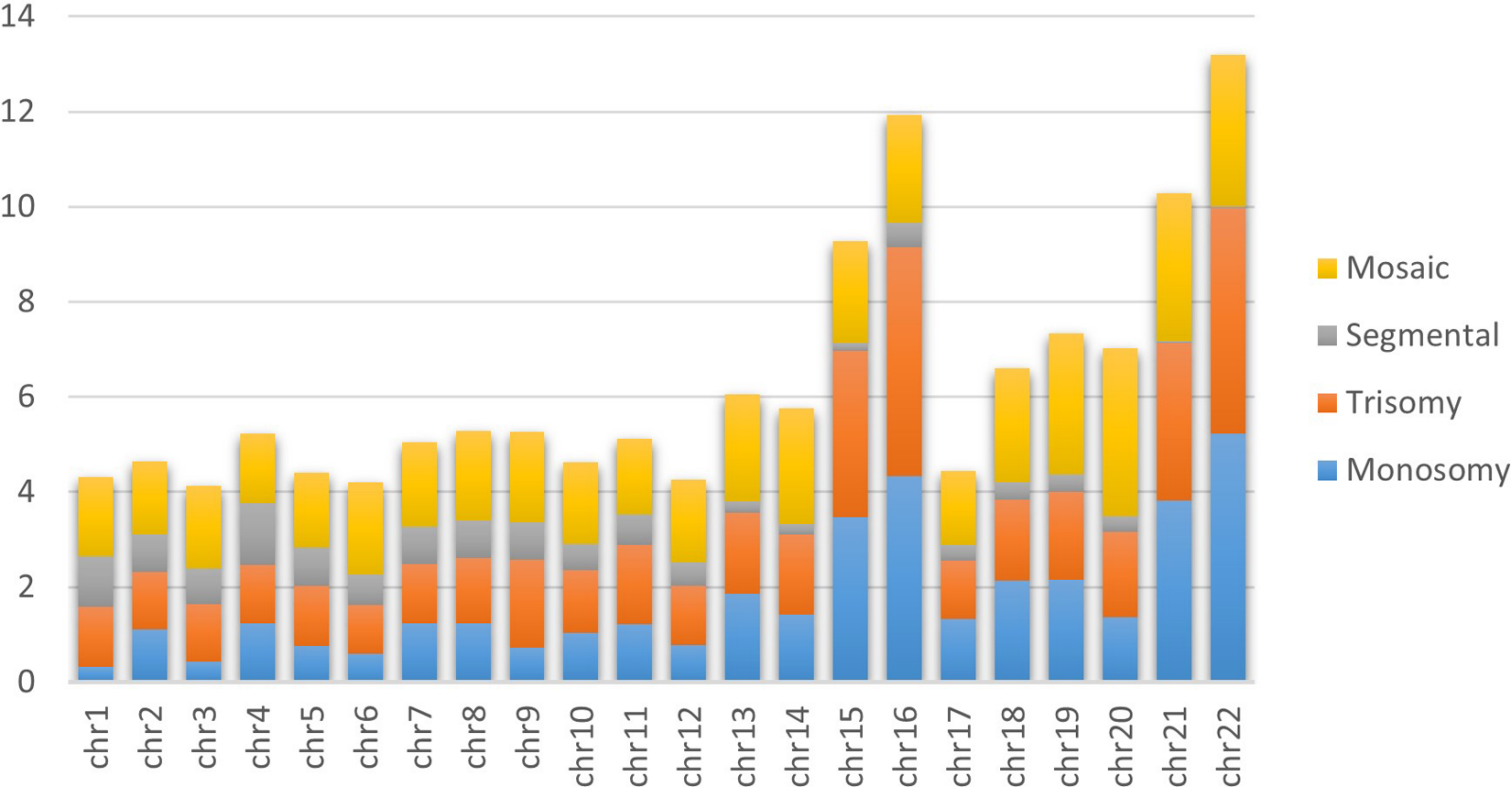
Incidence of aneuploidies per chromosome. The bar graph of aneuploidies demonstrates the aneuploidy distribution of 105,752 informative embryos for each chromosome. Yellow, grey, orange and blue represent the incidence of mosaic, segmental, uniform trisomy and uniform monosomy aneuploidy, respectively.

We analyzed 1,06,777 trophectoderm biopsies from 166 IVF clinics/centers across Brazil from 2019 to 2022, with 1,05,752 providing informative data. Table 1 describes the distribution of informative trophectoderm biopsy results (39.78% euploid and 60.22% aneuploid). We assessed the distribution of each aneuploid category separately—we found monosomy as the most common (16.96%), followed by trisomy (14.22%), mosaic aneuploidy (6.99%), and segmental aneuploidy (5.30%). Figure 1 depicts the proportion of monosomy, trisomy, segmental aneuploidies, and mosaic aneuploidies per chromosome of informative trophectoderm biopsies, where each bar stands for one chromosome, and a colored section of the bar represents the proportion of aneuploidy. Chromosomes 22 and 16 had the highest proportion of monosomy and trisomy, respectively. We observed segmental aneuploidy in higher proportions on chromosome 4, followed by chromosome 1; meanwhile, we observed the majority of mosaic aneuploidy present on the last three chromosomes (20, 22, and 21, in that order).

**Table 1:** Overview of informative trophectoderm embryo biopsies results by NGS.

Timeframe	2019–2022
No. of Centers	166
No. of Embryos analyzed	1,06,777
No. of informative embryos	1,05,752
Mean maternal age (SD)	37.91 (3.94)
Mean paternal age (SD)	40.12 (6.28)
Euploid embryos (%)	42,073 (39.78)
Aneuploid embryos (%)	63,679 (60.22)
Monosomy only (%)	17,939 (16.96)
Trisomy only (%)	15,042 (14.22)
Monosomy + trisomy (%)	8,309 (7.86)
Segmental aneuploidy only (%)	5,600 (5.30)
Duplication (%)	1,825 (1.73)
Deletion (%)	3,352 (3.17)
Duplication + deletion (%)	423 (0.40)
Mosaic aneuploidy (%)	7,392 (6.99)
Low mosaic aneuploidy (%)	5,191 (4.91)
High mosaic aneuploidy (%)	2,201 (2.08)
Multiple aneuploidies	9,397 (8.89)

Euploid: no chromosomal abnormality observed; Whole uniform aneuploidy: at least one aneuploidy for a whole chromosome observed; Segmental aneuploidy: only partial deletion/duplications observed; Low mosaicism: one or two low mosaic degree aneuploidies without additional uniform or segmental aneuploidies observed; High mosaicism: one or two high mosaic degree aneuploidies or one low and one high mosaic degree aneuploidies without additional uniform or segmental aneuploidies observed; multiple aneuploidies: combination of two or more of the categories described.

### Analysis dataset description

Applying the criteria described in the method section left 60,369 trophectoderm biopsies (defined as the analysis set) meeting the selection criteria for all variables (maternal age, embryo origin, day of blastocyst biopsy, morphology, and clinical indication). This analysis set contained 26,718 euploid trophectoderm biopsies, 3,177 with low mosaicism, 1,463 with high mosaicism, 3,046 with segmental aneuploidies, and 25,965 with uniform whole aneuploidies. Table 2 describes all the data presented in this section.

**Table 2:** Comparison of clinical conditions of PGT-A tests among different biopsy outcome types.

	Euploid (*n* = 26,718)	Low mosaic aneuploidy (*n* = 3,177)	High mosaic aneuploidy (*n* = 1,463)	Segmental (*n* = 3,046)	Whole uniform aneuploidy (*n* = 25,965)	Total (*n* = 60,369)
**Mean maternal age (SD)**	36.21 (3.50)*,β	36.41 (3.54)β	37.38 (3.30)α	36.48 (3.50)β	38.43 (2.90)α^,^β	37.22 (3.42)
**Mean paternal age (SD)**	39.07 (6.05)β	39.35 (6.11)	39.80 (5.90)	39.39 (6.04)	40.40 (5.84)ω^,^α	39.69 (5.99)
**Origin (%)**						
Fresh oocyte (FO)	89.15β^,^α	88.20β	89.47α	87.46β	90.38β^,^α	89.55
Vitrified blastocyst (VB)	5.88β^,^α	6.01β	5.13α	6.11β	5.03β^,^α	5.51
Vitrified oocyte (VO)	3.82β^,^α	4.60β	3.62α	5.29β	3.38β^,^α	3.74
Vitrified embryo (VE)	1.15β^,^α	1.20	1.78	1.15	1.21β^,^α	1.19
**Biopsy day (%)**						
D5	70.39β^,^α	64.78β	63.23α	59.06β^,^ϕ	59.55β^,^α	64.69
D6	27.95β^,^α	32.96β	33.22	38.28β	37.65β^,^α	33.04
D7	1.65β^,^α	2.27	3.55	2.66	2.80β^,^α	2.27
**Embryo sex (%)**						
Male	50.34β^,^α	48.80β	49.18α	51.96β	51.17β^,^α	50.66
Female	49.66β^,^α	51.20β	50.82α	48.04β^,^ϕ	48.83β^,^α	49.34
**Morphology**						
**Expansion (%)**						
2	1.95β^,^α	2.01	2.12	2.00	2.40β^,^α	2.15
3	35.88β^,^α	38.87β	43.27α	42.74β	43.32β^,^α	38.80
4	34.05β^,^α	33.36β	31.58α	33.52β	32.68β^,^α	33.34
5	24.12β^,^α	21.34β	19.28α	18.22β	18.69β^,^α	21.22
6	4.00β^,^α	4.41β	3.76α	3.51ω	2.82β^,^α	3.49
**Internal mass cell (%)**						
A	47.74β^,^α	37.61β	33.83α	33.62β^,^*	34.44β^,^α	40.44
B	40.84β^,^α	45.70β	46.68α	46.82β	46.96β^,^α	44.17
C	11.42β^,^α	16.69β	19.48α	19.57β	18.59β^,^α	15.38
D	0	0.09	0	0	0.01	0.01
**Trophectoderm (%)**						
A	37.96β^,^α	26.63β	21.31α	22.42β^,^ϕ	23.36β^,^α	29.89
B	44.39β^,^α	45.36β	47.03α	45.60β	45.33β^,^α	44.97
C	17.64β^,^α	27.98β	31.78α	31.98β	31.30β^,^α	25.13
D	0	0.03	0	0	0.01	0.01
**Indication (%)**						
Advanced Maternal Age (AMA)	25.58β^,^α	28.39β	36.77α	28.66β	44.15β^,^α	34.14
Aneuploidy Screening (AS)	25.62β^,^α	25.84β	19.00α	26.59β	14.19β^,^α	20.87
Unknown (UNK)	26.13β^,^α	23.61β	22.15α	22.55β	19.09β^,^α	22.69
Male factor (MF)	5.85β^,^α	5.07β	4.37α	5.71β	3.61β^,^α	4.80
Implantation Failure (IF)	2.86β^,^α	2.58β	2.39α	2.89β	1.90β^,^α	2.42
Uterus/Ovarian Factor (UOF)	1.33β^,^α	1.32β	0.75α	1.08β	1.89β^,^α	1.55
Pregnancy Loss (PL)	3.88β^,^α	4.00β	3.90α	3.35β	3.49β^,^α	3.69
Endometriosis (EN)	0.84β^,^α	0.72	0.55	0.62	0.83β^,^α	0.81
More than one indication (MIX)	7.44β^,^α	8.06β	9.64α	8.14β	9.98β^,^α	8.66
Others	0.47β^,^α	0.41	0.48	0.39	0.25β^,^α	0.37

* *p*-value < 0.05 when compared to low mosaicism.

^ϕ^ *p*-value < 0.01 when compared to low mosaicism

^ω^ *p*-value < 0.01 when compared to high mosaicism.

^α^ *p*-value < 0.001 when compared to low mosaicism.

^β^ *p*-value < 0.001 when compared to high mosaicism.

We observed a mean maternal age of 37.22 years old, with statistically significant differences between low mosaic trophectoderm biopsies and all groups except segmental aneuploidies. High mosaic trophectoderm biopsies also displayed significant differences in maternal age compared to all other groups. We observed a mean paternal age of 39.69, which presented significant differences between low mosaicism and whole aneuploidy; high mosaicism presented the same significant difference to euploidy. Analysis of embryo origin revealed that most trophectoderm biopsies derived from using FOs (89.55%). We observed a significant difference between low mosaicism and all other groups except segmental aneuploidy, while high mosaicism displayed significant differences from all other groups. Day-5 was the most common day of blastocyst biopsy (64.69%), presenting a significant difference between low and high mosaicism compared to all other groups. For day 6, we observed significant differences for high mosaicism compared to all other groups and for low mosaicism compared to euploid biopsies and biopsies with whole uniform aneuploidies. Day-7 presented a significant difference between low and high mosaicism compared to euploid and whole uniform aneuploidy. The proportions of embryo sex displayed significant differences between low and high mosaicism compared to all other groups. When analyzing morphology, we found expansion present primarily in grade 3 in all groups and significant differences of expansion grades across all groups except for grades 2 and 6. The inner cell mass morphologies were grade A for most euploid embryos, while grade B was the most observed for aneuploid embryos. We found significant differences in proportions in low and high mosaicisms compared to all groups (except segmental, which was not significantly different from high mosaicism for grades B and C). We did not evaluate Grade D due to insufficient observations. We found trophectoderm morphology classification grade B as the most common in all groups; we observed a significant difference in proportion compared to inner cell mass morphology. Again, we did not evaluate Grade D due to insufficient observations. AMA represented the most present clinical indication in our analysis set, followed by unknown and AS, with significant differences observed across all groups except between low mosaicism and segmental.

### Mosaicism and chromosomes

**Figure 2:**
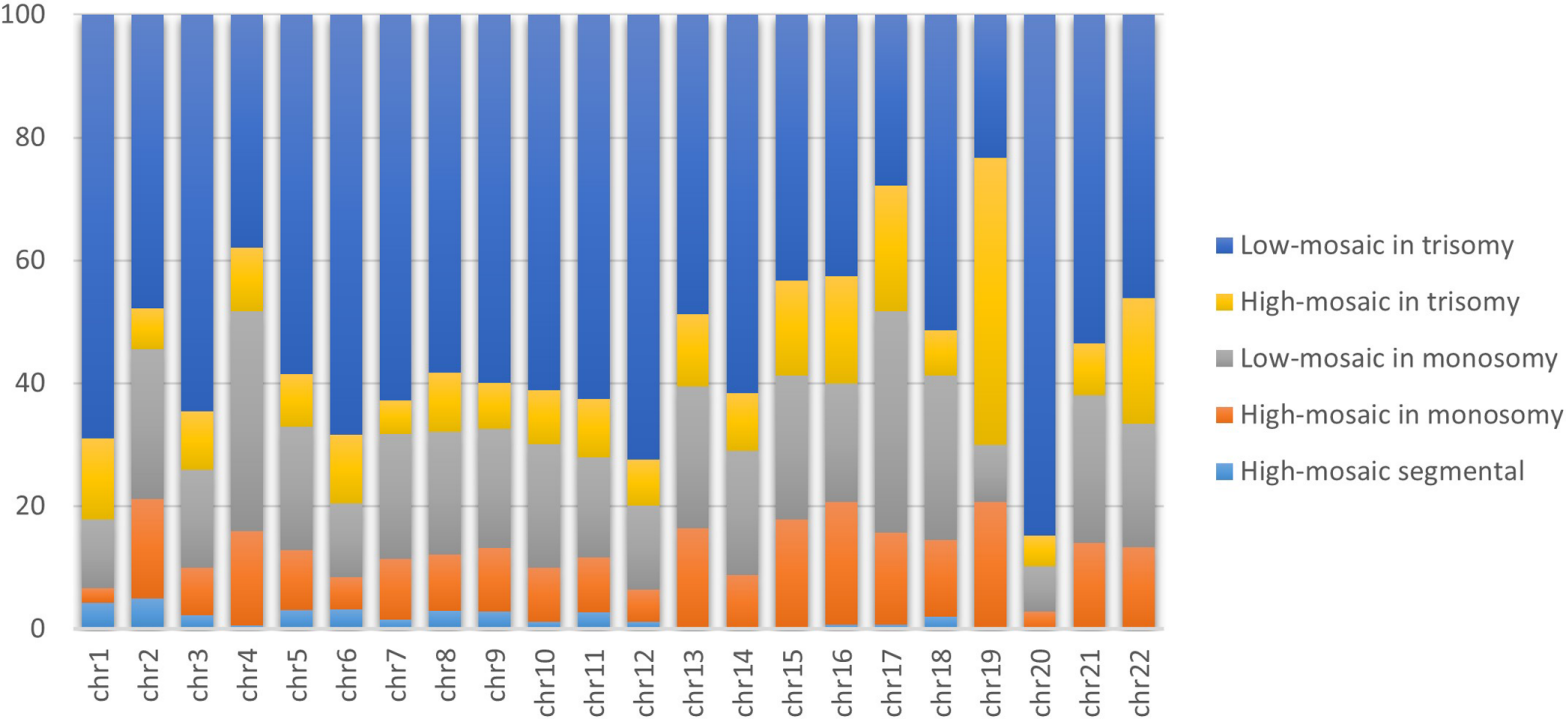
Proportion of distinct types of mosaicism per chromosome. The bar graph demonstrates the proportions of 4,640 mosaic trophectoderm biopsies subclassified into uniform or segmental chromosome mosaicism, stratified by the impact level (low and high). Orange, yellow, and light blue represent the proportions of high mosaic in monosomy, trisomy and segmental, respectively. While grey and light blue represent low mosaic in monosomy and trisomy, respectively.

Analysis of 4,640 embryos presenting only mosaicism revealed that 78.06% had only one chromosome affected with mosaicism, 21.91% had two chromosomes affected, and 0.02% had three or more chromosomes affected (Figure 2). We also assessed the distinct types of mosaicism observed by chromosome, observing low mosaicism in trisomy as overall the most common, followed by low mosaicism in monosomy. We found segmental high mosaicism present primarily in chromosomes 1 and 2, followed by chromosomes 6 and 5. We observed high mosaicism in monosomy as most common on chromosome 19, followed by chromosomes 16, 15, and 13. High mosaic in trisomy appeared mostly on chromosome 19, followed by chromosomes 22 and 17. Finally, we observed the lowest mosaicism in monosomy on chromosomes 17 and 4 (followed by chromosomes 18 and 2) and low mosaic in trisomy primarily present on chromosomes 20 (followed by chromosomes 12 and 1).

### Mosaicism according to clinic, center and operator

To visualize the proportion of mosaic cases per IVF clinic/center and per operator (embryologist), we selected clinics with 100 or more trophectoderm biopsies with diagnosed mosaicism and operators with 70 or more trophectoderm biopsies performed with diagnosed mosaicism (Tables 3, 4, respectively). The percentage of mosaicism varied from 5.89% to 8.43% among clinics, while the percentage of mosaicism varied from 5.12% to 8.85% when comparing operators. When looking into high and low mosaicism, the rate of high mosaicism varied from 0.87% to 2.5% among clinics and from 1.23% to 2.29% among different biopsy operators. For low mosaicism, clinics presented a variation in its percentage from 3.17% to 6.93%, while low mosaicism rates varied from 3.23% to 7.07% when comparing operators.

**Table 3:** Incidence of high and low mosaicism by IVF clinic/center.

Clinic	*N* biopsies	*N* biopsies with Mosaicism	% Mosaicism	% High mosaicism	% Low mosaicism
A	11,217	588	5.24	2.02	3.22
B	8,772	429	4.89	1.72	3.17
C	5,723	363	6.34	2.03	4.31
D	3,741	273	7.30	2.50	4.80
E	4,174	261	6.25	2.20	4.05
F	4,318	247	5.72	1.85	3.87
G	3,934	220	5.59	1.82	3.77
H	3,430	215	6.27	1.71	4.56
I	2,808	148	5.27	1.83	3.44
J	2,209	131	5.93	0.87	4.06
K	2,513	127	5.05	2.12	5.38
L	1,495	126	8.43	1.50	6.93
M	2,432	124	5.10	1.80	3.30
N	1,987	108	5.44	1.64	3.80

**Table 4:** Incidence of high and low mosaicism by operator.

Operator	*N* biopsies	*N* biopsies with Mosaicism	% Mosaicism	% High mosaicism	% Low mosaicism
1	10,042	602	5.99	1.96	4.03
2	4,332	240	5.54	1.70	3.84
3	2,119	147	6.94	2.07	4.87
4	2,260	123	5.44	1.68	3.76
5	2,343	120	5.12	1.89	3.23
6	1,652	111	6.72	2.21	4.51
7	2,032	116	5.71	1.77	3.94
8	1,821	115	6.32	2.00	4.32
9	1,209	107	8.85	1.78	7.07
10	1,622	94	5.80	1.77	4.03
11	1,221	88	7.21	2.29	4.92
12	1,298	87	6.70	1.73	4.97
13	1,273	81	6.36	1.68	4.68
14	1,263	78	6.18	2.38	3.80
15	1,430	76	5.31	1.23	4.08

### Associations of low mosaicism with clinical characteristics

We adjusted four models with low mosaicism as the dependent variable in comparison with (1) euploid, (2) segmental aneuploidy, (3) whole uniform aneuploidy, and (4) high mosaicism. We adjusted all models for maternal and paternal age, embryo origin, the day of blastocyst growth, embryo sex, morphology, and clinical indication. Tables 5, 6 detail the complete results of these analyses. The detailed outcome of the models considering interactions of maternal age with indication, and biopsy day with morphology can be accessed in Supplementary Table S4.

**Table 5:** Multiple logistic regression models for low and high mosaic aneuploidies from trophectoderm biopsies.

	Odds ratio (IC 95%)					
	Low mosaic aneuploidy^α^			High mosaic aneuploidy^α^		
	Euploid	Segmental	Whole Uniform aneuploidy	Euploid	Segmental	Whole Uniform aneuploidy
**Maternal age**	1.00 (0.99–1.02)	0.99 (0.97–1.01)	0.84 (0.83–0.85)^α^	1.09 (1.07–1.11)β	1.07 (1.04–1.10)^β^	0.89 (0.88–0.91)^β^
**Paternal age**	1.01 (1.00–1.01)	1.00 (0.99–1.01)	1.00 (1.00–1.01)	1.00 (0.99–1.01)	1.00 (0.99–1.01)	1.00 (0.99–1.01)
**Origin (vs. fresh oocyte)**						
Vitrified blastocyst	1.01 (0.86–1.17)	0.98 (0.79–1.21)	1.11 (0.94–1.31)	0.82 (0.64–1.04)	0.88 (0.66–1.17)	0.97 (0.75–1.22)
Vitrified oocyte	1.14 (0.95–1.36)	0.90 (0.71–1.14)	1.56 (1.29–1.88)^α^	0.78 (0.58–1.02)	0.60 (0.43–0.83)^ω^	1.15 (0.86–1.52)
Vitrified embryo	0.94 (0.66–1.31)	1.04 (0.65–1.66)	1.19 (0.83–1.65)	1.20 (0.78–1.78)	1.32 (0.78–2.21)	1.52 (0.99–2.23)^†^
**Biopsy day (vs. D5)**						
D6	1.11 (1.02–1.20)*	0.78 (0.70–0.88)^α^	0.77 (0.70–0.84)^α^	1.11 (0.98–1.25)	0.81 (0.70–0.93)^ω^	0.77 (0.68–0.88)^β^
D7	1.10 (0.84–1.42)	0.79 (0.56–1.11)	0.68 (0.52–0.87)^ϕ^	1.68 (1.22–2.26)^ω^	1.22 (0.83–1.78)	1.04 (0.76–1.39)
**Embryo sex (vs. female)**						
Male	0.97 (0.90–1.04)	0.87 (0.79–0.96)^ϕ^	0.89 (0.83–0.96)^ϕ^	1.00 (0.90–1.11)	0.91 (0.80–1.03)	0.92 (0.83–1.03)
**Morphology**						
**Expansion (vs. 3)**						
2	0.91 (0.69–1.19)	1.07 (0.74–1.54)	0.96 (0.73–1.25)	0.82 (0.55–1.17)	1.04 (0.66–1.63)	0.89 (0.60–1.27)
4	0.99 (0.90 1.08)	1.10 (0.97–1.24)^α^	1.16 (1.06–1.27)ϕ	0.87 (0.77–0.99)^†^	0.95 (0.82–1.11)	1.02 (0.90–1.16)
5	0.93 (0.84 1.03)	1.29 (1.12–1.49)^ϕ^	1.31 (1.18–1.46)^α^	0.79 (0.68–0.92)^ω^	1.10 (0.92–1.31)	1.10 (0.95–1.28)
6	1.07 (0.88 1.29)	1.54 (1.17–2.03)	1.94 (1.57–2.38)^α^	0.78 (0.58–1.04)	1.14 (0.79–1.62)	1.48 (1.09–1.98)^†^
**Inner mass cell (vs. A)**						
B	1.07 (0.97–1.18)	1.02 (0.89–1.16)	1.04 (0.94–1.15)	1.04 (0.91–1.20)	0.98 (0.82–1.16)	1.01 (0.87–1.16)
C	1.09 (0.94–1.26)	0.95 (0.78–1.15)	1.04 (0.90–1.20)	1.15 (0.94–1.40)	0.96 (0.76–1.22)	1.09 (0.89–1.33)
**Trophectoderm (vs. A)**						
B	1.37 (1.24–1.52)α	0.87 (0.75–1.01)	0.92 (0.82–1.02)	1.73 (1.49–2.02)^β^	1.10 (0.91–1.32)	1.18 (1.01–1.38)^†^
C	2.03 (1.78–2.32)α	0.84 (0.70–1.01)	0.90 (0.78 1.03)	2.69 (2.22–3.27)^β^	1.13 (0.89–1.43)	1.20 (0.98–1.45)
**Indication (vs. AMA)**						
Aneuploidy screening (AS)	0.94 (0.84–1.06)	0.97 (0.83–1.15)	1.24 (1.10–1.40)^α^	0.76 (0.64–0.90)ω	0.78 (0.63–0.96)^†^	0.96 (0.80–1.14)
Unknown (UNK)	0.85 (0.76 0.95)^ϕ^	1.03 (0.88–1.20)	1.09 (0.97–1.22)	0.82 (0.70–0.96)^†^	0.95 (0.79–1.15)	1.01 (0.86–1.18)
Male factor (MF)	0.81 (0.67–0.98)*	0.86 (0.67–1.10)	1.00 (0.81–1.21)	0.78 (0.58–1.03)	0.80 (0.57–1.12)	0.92 (0.69–1.22)
Implantation failure (IF)	0.82 (0.64–1.05)	0.93 (0.66–1.29)	1.06 (0.81–1.37)	0.82 (0.56–1.16)	0.87 (0.56–1.32)	1.04 (0.71–1.48)
Uterus/Ovarian factor (UOF)	0.99 (0.70–1.36)	1.14 (0.71–1.85)	0.86 (0.61–1.18)	0.52 (0.26–0.91)^†^	0.61 (0.29–1.19)	0.43 (0.22–0.76)^ω^
Pregnancy loss (PL)	0.98 (0.79–1.19)*	1.12 (0.84–1.49)	1.07 (0.86–1.31)	0.96 (0.71–1.27)	1.08 (0.75–1.53)	1.00 (0.74–1.33)
Endometriosis (EN)	0.84 (0.53–1.28)	1.05 (0.57–1.98)	0.88 (0.55–1.34)	0.62 (0.28–1.19)	0.74 (0.30–1.67)	0.61 (0.27–1.17)
More than one indication (MIX)	0.99 (0.85–1.15)	1.00 (0.82–1.23)	1.06 (0.92–1.23)	1.03 (0.84–1.25)	1.03 (0.81–1.31)	1.06 (0.87–1.29)
Others	0.80 (0.43–1.38)	1.10 (0.49–2.55)	1.23 (0.63–2.21)	0.97 (0.40–1.96)	1.44 (0.52–3.75)	1.50 (0.62–3.11)

Models adjusted to demonstrate the association between low and high mosaicism with clinical characteristics: low mosaic aneuploidy vs. euploidy, low mosaic aneuploidy vs. segmental aneuploidy, low mosaic aneuploidy vs. whole uniform aneuploidy, high mosaic aneuploidy vs. euploidy, high mosaic aneuploidy vs. segmental aneuploidy, high mosaicism aneuploidy vs. whole uniform aneuploidy and low mosaicism aneuploidy vs. high mosaicism aneuploidy. Analysis stratified by the outcome.

^α^ Reference category.

* *p*-value < 0.05 when compared to low mosaicism.

^†^ *p*-value < 0.05 when compared to high mosaicism.

^ϕ^ *p*-value < 0.01 when compared to low mosaicism.

^ω^ *p*-value < 0.01 when compared to high mosaicism.

^α^ *p*-value < 0.001 when compared to low mosaicism.

^β^ *p*-value < 0.001 when compared to high mosaicism.

**Table 6:** Multiple logistic regression model from trophectoderm biopsies to access the likelihood of an embryo being low mosaic compared to high mosaic.

	Odds ratio (IC 95%)
**Maternal age**	0.93 (0.91–0.95)^α^
**Paternal age**	1.00 (0.99–1.01)
**Origin (vs. fresh oocyte)**	
Vitrified blastocyst	1.17 (0.88–1.55)
Vitrified oocyte	1.45 (1.05–2.03)*
Vitrified embryo	0.80 (0.48–1.34)
**Biopsy day (vs. D5)**	
D6	0.96 (0.83–1.11)
D7	0.61 (0.42–0.91)*
**Sex embryo (vs. female)**	
Male	0.95 (0.83–1.07)
**Morphology**	
**Expansion (vs. 3)**	
2	1.05 (0.68–1.67)
4	1.14 (0.98–1.33)
5	1.20 (1.00–1.43)*
6	1.39 (0.99–1.98)
**Internal mass cell (vs. A)**	
B	1.03 (0.87–1.22)
C	0.98 (0.77–1.24)
**Trophectoderm (vs. A)**	
B	0.76 (0.64–0.91)^ϕ^
C	0.74 (0.59–0.93)*
**Indication (vs. AMA)**	
Aneuploidy screening (AS)	1.29 (1.05–1.58)*
Unknown (UNK)	1.07 (0.88–1.29)
Male fator (MF)	1.09 (0.78–1.52)
Implantation failure (IF)	1.02 (0.67–1.58)
Uterus/Ovarian factor (UOF)	1.90 (1.00–3.94)
Pregnancy loss (PL)	1.06 (0.76–1.50)
Endometriose (EN)	1.52 (0.70–3.66)
More than one indication (MIX)	1.01 (0.80–1.29)
Others	0.85 (0.34–2.30)

* *p*-value < 0.05.

^ϕ^ *p*-value < 0.01.

^α^ *p*-value < 0.001.

We found that every 1-year increase in maternal age reduced the chance of low mosaicism compared to whole uniform aneuploidy by 16% (Table 5). When comparing with high mosaicism, every 1-year increase in maternal age, reduce the chance of low mosaicism by 7% (Table 6) A vitrified oocyte as the embryo origin increased the chance of low mosaicism instead of whole uniform aneuploidy by 56% compared with fresh oocyte origin (Table 5). Similarly, a vitrified oocyte increased the chance of low mosaicism instead of high mosaicism by 45% (Table 6). A day-6 biopsy increased the risk of low mosaicism vs. euploidy compared to a day-5 biopsy; however, trophectoderm biopsies from day-6 are less likely to display low mosaicism than segmental or whole uniform aneuploidy (Table 5), and biopsies from day-7 are less likely to be low mosaic than high mosaic compared to day-5 biopsies, suggesting that day-5 biopsies have a better overall outcome. When adding interactions to the models, the biopsy day effects mainly the incidence of euploid embryos, embryos with whole uniform aneuploidy, but without significance for high mosaic (Supplementary Table S4).

We found male embryos less likely to display low mosaicism than segmental (13%) and whole aneuploidy (11%) compared to female embryos. At the morphology level, we observed a higher likelihood of low mosaicism than segmental and whole uniform aneuploidy in trophectoderm biopsies with expansion grades 4 and 5 (Table 5), while only grade 5 was more likely to have low mosaic than high mosaic (Table 6) compared to grade 3. Samples with trophectoderm morphology grade B had a 37% higher chance of possessing low mosaicism than euploidy (Table 5), and 24% lower chance of having low mosaicism than high mosaicism (Table 6) compared to grade A; furthermore, trophectoderm grade C had double chance of displaying low mosaicism than euploidy (Table 5), and 26% lower chance of displaying low mosaicism than high mosaicism (Table 6), compared to grade A. Regarding indications, compared to advanced maternal age, using aneuploidy screening as the clinical indication for the PGT-A test provided a 24% more likely chance of displaying low mosaicism than whole uniform aneuploidy (Table 5), and 29% higher chance of displaying low mosaicism than high mosaicism (Table 6). Finally, male factor indication reduces the chances of low mosaicism instead of euploidy by 19% compared to advanced maternal age.

When adding interactions to the models, the effects of indication regarding euploid and high mosaic had a loss of significance, similarly, the effects for expansion grade 4 regarding segmental and expansion grade 5, trophectoderm grade C and indication AS regarding high mosaic, also had a loss of significance (Supplementary Table S4). These results indicate a collinear effect on those variables.

### Associations of high mosaicism with clinical characteristics

We next adjusted three models with high mosaicism as the dependent variable in comparison with (1) euploid, (2) segmental aneuploidy, and (3) whole aneuploidy. We adjusted all models for maternal and paternal age, embryo origin, the day of blastocyst growth, embryo sex, morphology, and clinical indication. Table 5 details the complete results of this analysis. The outcomes of the models considering interactions are shown in Supplementary Table S4. It wasn't possible to adjust the model high mosaicism vs. segmental aneuploidy including interactions due to insufficient observations in these subgroups to perform the analysis.

We found that increased maternal age associated with an increased chance of high mosaicism instead of euploidy or segmental aneuploidy; however, an increase in maternal age associated with a lower likelihood of high mosaicism when comparing high mosaicism and whole uniform aneuploidy. We observed a 40% lower chance of high mosaicism instead of segmental aneuploidy for vitrified oocytes compared to fresh oocytes as the origin. Vitrified embryos as the origin associated with a 52% higher likelihood of high mosaicism instead of whole uniform aneuploidy. Biopsy on day 6 prompted a lower likelihood of high mosaicism instead of segmental (19%) or whole uniform aneuploidy (23%) than biopsy on day 5. Trophectoderm biopsies from day 7 possessed a 68% higher chance of high mosaicism than euploidy compared to day 5. At the morphological level, trophectoderm biopsies with expansion grades 4 and 5 display a lower likelihood of high mosaicism than euploid compared to grade 3 expansion. Trophectoderm morphology grades B and C displayed an increased likelihood than A of having high mosaicism instead of euploid than grade A. Using AS or UOF as the clinical indication for the PGT-A test provides a lower likelihood of high mosaicism than euploidy compared to using AMA. When comparing high mosaicism and segmental aneuploidy, using AS as the clinical indication for the PGT-A test reduces the chances of high mosaicism by 22% compared to using AMA.

When adding interactions to the models, the biopsy day, expansion grade 4 and indication effects regarding euploid, had a loss of significance. Similarly, the effects for vitrified embryo, expansion grade 6 and indication regarding whole uniform aneuploidy also had a loss of significance (Supplementary Table S4). These results indicate a collinear effect on those variables.

It is important to mention that, although our database is big, some groups inside the categories had a small number of observations, meaning a higher variability within the data. This situation causes wider confidence intervals with a larger margin of error for the analysis involving such groups, even though significance was reached.

## Discussion

After selecting those samples that fulfilled all criteria described in the methods section (maternal age, embryo origin, day of blastocyst biopsy, morphology, and clinical indication), we obtained a higher incidence of euploidy followed by whole uniform aneuploidy, low mosaicism, segmental aneuploidy and high mosaicism, respectively. The incidence proportions encountered in our study agree with the frequencies previously described by Rodrigo et al. (3), finding a 6.99% rate of mosaicism in this study. In assisted reproduction treatments, rates of mosaicism found in IVF are considered small (around 4%–5% of trophectoderm biopsy) and do not significantly affect the diagnosis of PGT-A (9).

We found that the incidence of whole uniform aneuploidy and mosaicism increased with decreasing chromosome size; mosaicism and complete aneuploidy appeared most often on chromosomes 13–22. Higher aneuploidy rates had previously been observed on chromosomes with sizes <90 Mbp (10), indicating that smaller-sized chromosomes had a greater chance of cell division errors. Inter-chromosomal heterogeneity can be explained by structural differences such as arm and centromere size (11); however, not only does size determine chromosomal alterations, but other factors (such as AMA, MF, and embryo quality) also contribute. We observed mosaicism most frequently on chromosomes 20, 22, and 19. Osman et al. reported chromosomes 22, 4, and 19 as most frequently displaying mosaicism (12), while Nakhuda et al. found the highest incidence on chromosomes 21, 22, and 2 (13), and Chuang et al. detected a higher prevalence on chromosomes 14, 1, and 9 (10). The reasons for the general lack of consensus on the chromosomes most frequently affected by mosaicism could derive from the methodologies used for PGT-A and the defined parameters used for the detection of mosaicism by the individual study centers.

Increasing maternal age influences the likelihood of embryonic aneuploidy. We found a slight but significant increase in mean maternal age between mosaicism and complete aneuploidy and a slight decrease between mosaicism and euploidy. Rodrigo et al. described higher rates of high-degree mosaicism and whole uniform aneuploidies for older female and male patients (3). In addition, only a slight decrease in mosaicism in women over 37 compared to younger patients had been previously detected (14).

Chromosomal mosaicism likely originates during the first embryonic cleavages by mitotic errors after fertilization (15), while whole aneuploidies originate from chromosome segregation errors during meiotic division for gamete formation (16). Maternal age alone may not represent the only factor associated with mosaicism; however, in the analysis of interaction and the logistic regression analysis, adjusted for all clinical characteristics, proved that age can have an isolated effect and for each 1-year increase in maternal age, the chance of low and high mosaic in relation to whole aneuploidy is reduced.

Referring to the origin of the embryo, the logistic regression analysis identified oocyte and embryo vitrification as a factor reducing the incidence of embryo whole uniform aneuploidy and increasing the probability of mosaicism. A result like this one was also found in another group of embryo samples (3). This effect may be a consequence of the impact caused by the vitrification process on embryo survival. Embryos that are less viable due to ploidy or morphology are less likely to reach the blastocyst stage after the thawing process.

The analysis of biopsy timing found day 5 as the most common choice among the groups; however, we observed significant differences in the proportions between low and high mosaicism compared to other groups. In addition to the logistic regression analysis, we also observed that day 5 possessed a better overall outcome than days 6 and 7, a finding consistent with decreased embryo quality at biopsy time. In fact, the evaluation of the interaction between the day of biopsy and embryo morphology showed that the two variables may not be independent.

We discovered differences in the distribution of embryo morphological classification among the euploid and aneuploid groups. Individual morphological components adopted in blastocyst evaluation (i.e., expansion degrees, inner cell mass grades, and trophectoderm grades) have been independently correlated with euploid/aneuploid status (17, 18). Considering the grade of expansion, grade 3 represented the most common value across all groups; however, we found significant differences in this distribution. Chen et al. indicated a greater likelihood of euploidy among blastocysts with good-quality inner mass cell or trophectoderm morphology (17). Similarly, most euploid embryos had grade A inner mass cell morphology, while grade B represented the most observed grade for aneuploidies. We observed grade B as the most common grade across all groups when studying trophectoderm morphology; however, grade B displayed a greater likelihood of high mosaicism instead of euploidy, and grade C had a greater likelihood of low or high mosaic instead of euploidy compared to grade A. Although our finding that blastocyst morphology related to the euploid/aneuploid status of embryonic biopsies reflects previously reported data well, we noted that variability in embryo grading might derive from factors such as subjective judgments of individual embryologists, laboratory settings, and observational time windows. Variations in morphological classification can make correlating the distribution of morphological categories between the euploid/aneuploid status a challenging task.

Mosaicism occurs due to mitotic errors during the first embryonic cleavages (19). Studies have revealed that IVF laboratory conditions can increase mosaicism rates due to exposure to environmental, mechanical, and chemical stressors (15, 20, 21). The higher occurrence of mitoses during the blastocyst stage prompts an increased error rate. We found an average level of mosaicism among operators in this study of 6.28%, with a variance of <3%. We found a relatively high variation in the mosaicism rate compared to Osman et al. (22), who obtained an average of 5.2% and a variation of 1%, when they considered, 4 embryologists performing embryo biopsies and 6 embryologists loading the samples into designated tubes. The greater number of clinics and embryologists considered in our study may be the reason why we found this high variation in the rate of mosaicism compared to Osman et al. Another factor that may influence the differences between our level of mosaicism among operators and the level found by Osman et al. is that the cited study did not consider the indications of these groups. Overall, we must consider all factors involved in diagnosing mosaicism, which can lead to the discarding of an embryo.

Patients should be informed of the benefits, risks, and limitations of PGT-A technology before pursuing this test (23, 24). Couples should be advised that PGT-A results do not provide results with 100% accuracy in their ability to diagnose the chromosomal status of an embryo, given that only trophectoderm cells undergo an evaluation. In pre-test counseling, some information should be provided, such as the frequency of mosaicism results by the laboratory, clinical and technical difficulties in interpreting results, and limited outcome data available regarding the transfer procedure, including congenital anomalies and other adverse perinatal outcomes (23, 25). In post-test counseling, the transfer of mosaic embryos could be discussed and considered by couples under certain circumstances, such as the lack of euploid embryos (after an IVF/PGT-A - with or without Preimplantation Genetic Testing for Monogenic Disorders (PGT-M) or Preimplantation Genetic Testing for Structural Rearrangements (PGT-SR)—cycle or prior to use of all) (23).

The data regarding mosaic embryo transfer outcomes also represents a challenge during post-test counseling. Unfortunately, in our laboratory, we have information about the clinical outcomes of each clinic in relation to mosaic embryo transfer of a minority of cases and for this reason, we do not have enough information in numbers to insert the transfer results of the mosaic embryos involved in this study. However, studies have been performed by distinct groups in order to help to understand the results and guide clinicians and patients. Capalbo and colleagues, in their multicenter non-selection prospective trial, transferred 484 euploid, 282 low mosaic (where 20%–30% of the cells are aneuploid) and 131 moderate mosaic (30%–50%) embryos. They found that low mosaics had a miscarriage rate of 11.0% and moderate mosaics 12.7% (26). Viotti and colleagues in their retrospective multicenter study compared the transfer of 1,000 mosaic embryos and over 5,500 euploid embryos between 2015 and 2020. They stratified the mosaics based on the specific type of abnormality, and whether they were <50% mosaic or >50% mosaic. They concluded mosaics can have a varying rate of miscarriage depending on the type of abnormality present (27).

Also, higher-level mosaics may carry an increased risk of adverse outcomes and are less favorable than lower-level mosaics; however, we still lack data in this sense. Overall, the percentage of mosaicism appears to be a more efficient success predictor than the specific chromosome affected (26). Viotti et al. and Capalbo et al. found that low-level mosaicism can have successful clinical outcomes (26, 27), therefore low mosaics should be considered with good potential for embryo transfer. Nevertheless, prenatal testing is recommended for any pregnancy after PGT-A or IVF (28, 29), and the decision should be made only by the couple after extensive genetic counseling (27–29).

## Data Availability

The data analyzed in this study is subject to the following licenses/restrictions: It belongs to Igenomix. Requests to access these datasets should be directed to marcia.riboldi@igenomix.com.
