# Supplementary material for: The embryo mosaicism profile of next-generation sequencing PGT-A in different clinical conditions and their associations
Source: Front Reprod Health. 2023 Mar 27;5:1132662. doi: 10.3389/frph.2023.1132662 (PMC10083245; doi:10.3389/frph.2023.1132662)
Supplement: Supplementary file 1 [file Table1.docx]

**The Embryo Mosaicism Profile of Next-Generation Sequencing PGT-A in Different Clinical Conditions and their Associations**

Hadassa Campos Heiser^1^, Natalia Fagundes Cagnin^1^, Mariane Uehara de Souza^1^, Taccyanna Mikulski Ali^1^, Paula R. Q. Estrada^1^, Camila C. W. D. de Souza ^1^, Bruno Coprerski^1^, Carmen Rubio^2^ and Marcia Riboldi^1, *^

**^1^** Igenomix Brasil, Laboratory of Genetic Medicine, São Paulo, Brazil

**^2^** Igenomix SLU, Valencia, Spain

***Correspondence**

Marcia Riboldi

email: [marcia.riboldi@](mailto:marcia.riboldi@)igenomix.com

**Keywords:** PGT-A, NGS, aneuploidy, mosaicism, IVF.

| **Table S1: Distribution of Outcomes per Chromosome from Trophectoderm Embryo Biopsies Performed by NGS** | | | | | | | | | | | | |
| --- | --- | --- | --- | --- | --- | --- | --- | --- | --- | --- | --- | --- |
| **Absolute values** | | | | | | |  | **Percentage %** | | | | |
| **Chromosome** | **Euploid** | **Monosomy** | **Trisomy** | **Segmental** | **Mosaic** | **Total** |  | **Euploid** | **Monosomy** | **Trisomy** | **Segmental** | **Mosaic** |
| **chr1** | 101035 | 328 | 1345 | 1111 | 1765 | 105584 |  | 95.69 | 0.31 | 1.27 | 1.05 | 1.67 |
| **chr2** | 100471 | 1164 | 1269 | 827 | 1620 | 105351 |  | 95.37 | 1.10 | 1.20 | 0.78 | 1.54 |
| **chr3** | 101269 | 456 | 1269 | 793 | 1836 | 105623 |  | 95.88 | 0.43 | 1.20 | 0.75 | 1.74 |
| **chr4** | 100097 | 1311 | 1298 | 1357 | 1557 | 105620 |  | 94.77 | 1.24 | 1.23 | 1.28 | 1.47 |
| **chr5** | 100954 | 809 | 1318 | 855 | 1677 | 105613 |  | 95.59 | 0.77 | 1.25 | 0.81 | 1.59 |
| **chr6** | 101094 | 618 | 1088 | 683 | 2035 | 105518 |  | 95.81 | 0.59 | 1.03 | 0.65 | 1.93 |
| **chr7** | 100312 | 1306 | 1313 | 832 | 1869 | 105632 |  | 94.96 | 1.24 | 1.24 | 0.79 | 1.77 |
| **chr8** | 100055 | 1297 | 1460 | 827 | 1995 | 105634 |  | 94.72 | 1.23 | 1.38 | 0.78 | 1.89 |
| **chr9** | 100080 | 759 | 1949 | 849 | 1993 | 105630 |  | 94.75 | 0.72 | 1.85 | 0.80 | 1.89 |
| **chr10** | 100772 | 1087 | 1394 | 591 | 1804 | 105648 |  | 95.38 | 1.03 | 1.32 | 0.56 | 1.71 |
| **chr11** | 100236 | 1286 | 1758 | 678 | 1686 | 105644 |  | 94.88 | 1.22 | 1.66 | 0.64 | 1.60 |
| **chr12** | 101165 | 826 | 1318 | 524 | 1819 | 105652 |  | 95.75 | 0.78 | 1.25 | 0.50 | 1.72 |
| **chr13** | 99269 | 1955 | 1804 | 263 | 2363 | 105654 |  | 93.96 | 1.85 | 1.71 | 0.25 | 2.24 |
| **chr14** | 99589 | 1501 | 1781 | 228 | 2577 | 105676 |  | 94.24 | 1.42 | 1.69 | 0.22 | 2.44 |
| **chr15** | 95869 | 3663 | 3694 | 176 | 2273 | 105675 |  | 90.72 | 3.47 | 3.50 | 0.17 | 2.15 |
| **chr16** | 93060 | 4575 | 5082 | 546 | 2400 | 105663 |  | 88.07 | 4.33 | 4.81 | 0.52 | 2.27 |
| **chr17** | 100973 | 1404 | 1301 | 342 | 1650 | 105670 |  | 95.56 | 1.33 | 1.23 | 0.32 | 1.56 |
| **chr18** | 98703 | 2257 | 1802 | 382 | 2536 | 105680 |  | 93.40 | 2.14 | 1.71 | 0.36 | 2.40 |
| **chr19** | 97952 | 2275 | 1950 | 384 | 3151 | 105712 |  | 92.66 | 2.15 | 1.84 | 0.36 | 2.98 |
| **chr20** | 98261 | 1441 | 1888 | 363 | 3730 | 105683 |  | 92.98 | 1.36 | 1.79 | 0.34 | 3.53 |
| **chr21** | 94840 | 4043 | 3491 | 46 | 3288 | 105708 |  | 89.72 | 3.82 | 3.30 | 0.04 | 3.11 |
| **chr22** | 91758 | 5524 | 5011 | 33 | 3385 | 105711 |  | 86.80 | 5.23 | 4.74 | 0.03 | 3.20 |

**SUPLEMENTAL MATERIAL**

| **Table S2: Distribution of the Origin of the Embryo According to the Result.** | | | | | |
| --- | --- | --- | --- | --- | --- |
| **Origin/Outcome** | **Euploid (n=26,718)** | **Low mosaic aneuploidy (n=3,177)** | **High mosaic aneuploidy (n=1,463)** | **Segmental (n=3,046)** | **Whole Uniform aneuploidy (n=25,965)** |
| Fresh oocyte (FO) | 44.06ᵝᵟ | 5.18ᵝ | 2.42ᵟ | 4.93ᵝ | 43.41ᵝᵟ |
| Vitrified blastocyst (VB) | 47.21ᵝᵟ | 5.74ᵝ | 2.25ᵟ | 5.59ᵝ | 39.21ᵝᵟ |
| Vitrified oocyte (VO) | 45.20ᵝᵟ | 6.46ᵝ | 2.35ᵟ | 7.13ᵝ | 38.87ᵝᵟ |
| Vitrified embryo (VE) | 42.50ᵝᵟ | 5.28 | 3.61 | 4.86 | 43.75ᵝᵟ |
| *p-value < 0.05 when compared to low mosaicism; †p-value < 0.05 when compared to high mosaicism | | | | | |
| ᵠp-value < 0.01 when compared to low mosaicism; ᶷp-value < 0.01 when compared to high mosaicism | | | | | |
| ᵟp-value < 0.001 when compared to low mosaicism; ᵝp-value < 0.001 when compared to high mosaicism  Analysis stratified by outcome | | | | | |

| **Table S3: Distribution of Types of Mosaicism per Chromosome from Trophectoderm Embryo Biopsies Performed by NGS** | | | | | | | | | | | | |
| --- | --- | --- | --- | --- | --- | --- | --- | --- | --- | --- | --- | --- |
| **Absolute values** | | | | | | |  | **Percentage %** | | | | |
| **Chromosome** | **High mosaic segmental** | **High mosaic in monosomy** | **Low mosaic in monosomy** | **High mosaic in trisomy** | **Low mosaic in trisomy** | **Total** |  | **High mosaic segmental** | **High mosaic in monosomy** | **Low mosaic in monosomy** | **High mosaic in trisomy** | **Low mosaic in trisomy** |
| **chr1** | 9 | 5 | 24 | 28 | 147 | 213 |  | 4.23 | 2.35 | 11.27 | 13.15 | 69.01 |
| **chr2** | 9 | 29 | 44 | 12 | 86 | 180 |  | 5.00 | 16.11 | 24.44 | 6.67 | 47.78 |
| **chr3** | 5 | 17 | 35 | 21 | 142 | 220 |  | 2.27 | 7.73 | 15.91 | 9.55 | 64.55 |
| **chr4** | 1 | 28 | 65 | 19 | 69 | 182 |  | 0.55 | 15.38 | 35.71 | 10.44 | 37.91 |
| **chr5** | 5 | 16 | 33 | 14 | 96 | 164 |  | 3.05 | 9.76 | 20.12 | 8.54 | 58.54 |
| **chr6** | 7 | 12 | 27 | 25 | 154 | 225 |  | 3.11 | 5.33 | 12.00 | 11.11 | 68.44 |
| **chr7** | 3 | 20 | 41 | 11 | 127 | 202 |  | 1.49 | 9.90 | 20.30 | 5.45 | 62.87 |
| **chr8** | 6 | 19 | 41 | 20 | 120 | 206 |  | 2.91 | 9.22 | 19.90 | 9.71 | 58.25 |
| **chr9** | 6 | 22 | 41 | 16 | 127 | 212 |  | 2.83 | 10.38 | 19.34 | 7.55 | 59.91 |
| **chr10** | 2 | 15 | 34 | 15 | 104 | 170 |  | 1.18 | 8.82 | 20.00 | 8.82 | 61.18 |
| **chr11** | 4 | 13 | 24 | 14 | 92 | 147 |  | 2.72 | 8.84 | 16.33 | 9.52 | 62.59 |
| **chr12** | 2 | 9 | 24 | 13 | 126 | 174 |  | 1.15 | 5.17 | 13.79 | 7.47 | 72.41 |
| **chr13** | 0 | 39 | 55 | 28 | 116 | 238 |  | 0.00 | 16.39 | 23.11 | 11.76 | 48.74 |
| **chr14** | 0 | 26 | 60 | 28 | 183 | 297 |  | 0.00 | 8.75 | 20.20 | 9.43 | 61.62 |
| **chr15** | 0 | 45 | 59 | 39 | 109 | 252 |  | 0.00 | 17.86 | 23.41 | 15.48 | 43.25 |
| **chr16** | 2 | 59 | 57 | 52 | 126 | 296 |  | 0.68 | 19.93 | 19.26 | 17.57 | 42.57 |
| **chr17** | 1 | 22 | 53 | 30 | 41 | 147 |  | 0.68 | 14.97 | 36.05 | 20.41 | 27.89 |
| **chr18** | 5 | 32 | 68 | 19 | 131 | 255 |  | 1.96 | 12.55 | 26.67 | 7.45 | 51.37 |
| **chr19** | 0 | 90 | 41 | 204 | 102 | 437 |  | 0.00 | 20.59 | 9.38 | 46.68 | 23.34 |
| **chr20** | 0 | 15 | 40 | 27 | 457 | 539 |  | 0.00 | 2.78 | 7.42 | 5.01 | 84.79 |
| **chr21** | 0 | 64 | 110 | 39 | 245 | 458 |  | 0.00 | 13.97 | 24.02 | 8.52 | 53.49 |
| **chr22** | 0 | 59 | 90 | 91 | 206 | 446 |  | 0.00 | 13.23 | 20.18 | 20.40 | 46.19 |

| **Table S4: Results of Multiple Logistic Regression Model from Trophectoderm Biopsies Including Interactions of Maternal Age with Indication and Biopsy Day with Morphology Grade (Expansion, Inner Mass Cell, Trophectoderm)** | | | |
| --- | --- | --- | --- |
| **Low mosaic x Euploid** | | **Low mosaic x Segmental** | **Low mosaic x Whole uniform aneuploidy** |
| Day 6: loss of significance | | Day 6: significance kept | Maternal age: significance kept |
| TE grades B and C: Significance kept | | Male: significance kept | Vitrified oocyte: significance kept |
| Indic. UNK and MF: loss of significance | | Exp. grade 4: loss of significance | Day 6 and 7: loss of significance |
| **Significant interactions: OR (IC 95%)** | | Exp. grade 5: significance kept | Male: significance kept |
| Day 6-Exp. grade 4: 1.23(1.01 - 1.50)* | | **Significant interactions: OR (IC 95%)** | Exp. grades 4, 5 and 6: significance kept |
| Day 7-TE grade B: 0.31(0.12 – 0.86)* | | Day 6-IMC grade B: 1.53(1.13 – 2.07)ᵠ | Indic. AS: significance kept |
| Day 7-TE grade C: 0.29(0.10 – 0.84)* | |  | **New significant associations: OR (IC 95%)** |
|  | |  | Indic. IF: 0.04(0.87 - 1.22)* |
|  | |  | Indic. MF: 0.01(0.00 - 0.10)ᵟ |
|  | |  | Indic. PL: 0.05(0.00 - 0.59)* |
|  | |  | Indic. UNK: 0.04(0.01 - 0.25)ᵟ |
|  | |  | **Significant interactions: OR (IC 95%)** |
|  | |  | Maternal age-Indic. AS: 1.11(1.06 - 1.17)ᵟ |
|  | |  | Maternal age-Indic. IF: 1.08(1.00 - 1.17)* |
|  | |  | Maternal age-Indic. MF: 1.13(1.06 - 1.20)ᵟ |
|  | |  | Maternal age-Indic. PL: 1.08(1.01 - 1.16)* |
|  | |  | Maternal age-Indic. UNK: 1.09(1.04 - 1.14)ᵟ |
|  | |  | Day 6-TE grade C: 0.72(0.53 - 0.97)* |
|  | |  | Day 7-IMC grade B: 4.26(1.25 - 20.29)* |
|  | |  | Day 7-TE grade B: 0.35(0.13 - 1.01)* |
|  | |  | Day 7-TE grade C: 0.23(0.08 - 0.71)ᵠ |
|  | |  |  |
| **High mosaic x Euploid** | | **High mosaic x Whole uniform aneuploidy** | **Low mosaic x High mosaic** |
| Maternal age: significance kept | | Maternal age: significance kept | Maternal age: significance kept |
| Day 7: loss of significance | | Vitrified embryo: loss of significance | Vitrified oocyte: significance kept |
| Exp. 4: loss of significance | | Day 6: loss of significance | Day 7: loss of significance |
| Exp. 5: significance kept | | Exp. grade 6: loss of significance | Exp. grade 5: loss of significance |
| TE grades B and C: significance kept | | TE grade B: significance kept | TE grade B: significance kept |
| Indic. AS, UNK and UOF: loss of significance | | Indic. UOF: loss of significance | TE grade C: loss of significance |
| **New significant associations: OR (IC 95%)** | | **New significant associations: OR (IC 95%)** | Indic. AS: loss of significance |
| IMC grade C: 1.32(1.01 - 1.70)† | | IMC grade C: 1.39(1.07 - 1.80)† | **Significant interactions: OR (IC 95%)** |
| **Significant interactions: OR (IC 95%)** | | Indic. UNK: 0.09(0.01 - 0.91)† | Day 7-IMC grade B: 7.16(1.23 - 63.14)* |
| Maternal age-Indic. AS: 0.93(0.87 - 1.00)† | | **Significant interactions: OR (IC 95%)** |  |
| Day 7-Exp. grade 6: 3.94(1.37 - 12.14)† | | Maternal age-Indic. UNK: 1.06(1.00 - 1.13)† |  |
|  | | Day 6-IMC grade C: 0.64(0.42 - 0.99)† |  |
| *p-value < 0.05 when compared to low mosaicism; †p-value < 0.05 when compared to high mosaicism | | | |
| ᵠp-value < 0.01 when compared to low mosaicism; ᶷp-value < 0.01 when compared to high mosaicism | | | |
| ᵟp-value < 0.001 when compared to low mosaicism; ᵝp-value < 0.001 when compared to high mosaicism | | | |
